# Current understanding and biotechnological application of the bacterial diterpene synthase CotB2

**DOI:** 10.3762/bjoc.15.228

**Published:** 2019-10-02

**Authors:** Ronja Driller, Daniel Garbe, Norbert Mehlmer, Monika Fuchs, Keren Raz, Dan Thomas Major, Thomas Brück, Bernhard Loll

**Affiliations:** 1 Institute of Chemistry and Biochemistry, Laboratory of Structural Biochemistry, Freie Universität Berlin, Takustr. 6, 14195 Berlin, Germany; 2 present address: Department of Molecular Biology and Genetics, Aarhus University, Gustav Wieds Vej 10, 8000 Aarhus C, Denmark; 3 present address: Danish Research Institute of Translational Neuroscience - DANDRITE, Nordic-EMBL Partnership for Molecular Medicine, Aarhus C, Denmark; 4 Werner Siemens Chair of Synthetic Biotechnology, Dept. of Chemistry, Technical University of Munich (TUM), Lichtenbergstr. 4, 85748 Garching, Germany; 5 Department of Chemistry, Bar-Ilan University, Ramat-Gan 52900, Israel

**Keywords:** biotechnology, CotB2, crystal structure, cyclooctatin, diterpene, reaction mechanism, terpene synthase

## Abstract

CotB2 catalyzes the first committed step in cyclooctatin biosynthesis of the soil bacterium *Streptomyces melanosporofaciens.* To date, CotB2 represents the best studied bacterial diterpene synthase. Its reaction mechanism has been addressed by isoptope labeling, targeted mutagenesis and theoretical computations in the gas phase, as well as full enzyme molecular dynamic simulations. By X-ray crystallography different snapshots of CotB2 from the open, inactive, to the closed, active conformation have been obtained in great detail, allowing us to draw detailed conclusions regarding the catalytic mechanism at the molecular level. Moreover, numerous alternative geranylgeranyl diphosphate cyclization products obtained by CotB2 mutagenesis have exciting applications for the sustainable production of high value bioactive substances.

## Introduction

Terpenes represent one of the most diverse groups of natural biomolecules [1–3]. Sesqui- and diterpenes are a diverse class of secondary metabolites derived predominantly from plants, marine invertebrates, fungi and some prokaryotes [4–8]. Properties of these natural products include antitumor, anti-oxidant, anti-inflammatory, antiviral, antimalarial, antibiotic, neuroprotective and even insecticidal activities, which makes these compounds high-value commercial targets for the chemical and pharmaceutical industry [9–10]. Structural diversity of diterpenes is created by the terpene synthase (TPS) enzyme family, which use acyclic isoprenoid precursors to generate a vast number of (poly)cyclic hydrocarbon scaffolds. Remarkably, this complex chemical reaction, comprising changes in bonding, hybridization as well as the introduction of specific stereochemistry, is performed in a single reaction cascade without consumption of a cofactor [11]. In this review we will focus particularly on bacterial diterpene synthases, in context with other sesqui- and ditperpene synthases of bacterial, fungal and plant origin.

**Figure 1:**
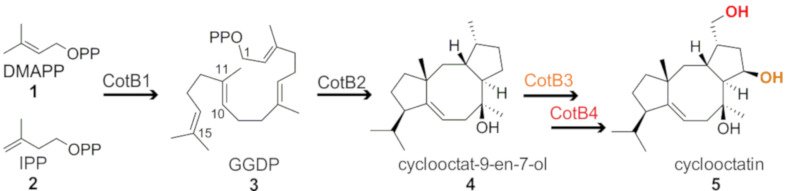
CotB1 synthesizes geranylgeranyl diphosphate (GGDP) **3** from the substrates dimethylallyl diphosphate (DMAPP) **1** and isopentenyl diphosphate (IPP) **2**. The acyclic substrate GGDP (**3**) is stereospecifically cyclized by CotB2 to cyclooctat-9-en-7-ol (**4**), with a fusicoccane 5–8–5 fused ring system. Two cytochrome P450 enzymes, CotB3 and CotB4, subsequently functionalize cyclooctat-9-en-7-ol (**4**) to the bioactive compound cyclooctatin (**5**).

The initial step in diterpene biosynthesis (Figure 1) is the incremental condensation of dimethylallyl diphosphate (**1**) and isopentylen diphosphate (**2**) [12] to the acyclic terpene synthase substrate geranylgeranyl diphosphate **3** (GGDP) [1,13–16]. Following initial substrate binding and folding in a product-like conformation, the cyclization reaction can be subdivided into three steps: (1) generation of a reactive allyl carbocation as a result of heterolytic cleavage of the pyrophosphate–hydrocarbon bond or protonation of a double bond, (2) propagation of the carbocation along the forming terpene skeleton as a result of ring formations, hydride and/or methyl shifts, de- and reprotonation of intermediates, the creation of a terminal carbocation (3) and finally the quenching of the carbocation by a base or water [16–17].

TPSs can be divided into two distinct classes, which are distinguished by their substrate activation mechanism. Whereas ionization of an isoprenoid diphosphate is caused by hydrolysis of the pyrophosphate by a trinuclear metal cluster in class I terpenoid cyclases, class II reactions proceed via a protonation of the terminal carbon–carbon double bond of an isoprenoid substrate [3]. In addition to the differences in the activation mechanism, the two classes of TPSs have an unrelated overall fold. Class I TPSs establish an α-helical bundle fold [18] and are structurally related to the isoprenyl diphosphate synthases such as the farnesyl (C15) or geranylgeranyl (C20) diphosphate synthase, which generate the substrates of sesqui- and diterpene synthases, respectively. In bacteria, diterpene synthases almost exclusively belong to class I TPSs. A few exceptions exist, for example the terpentedienyl-diphosphate synthase from *Kitasatospora griseola* [19], which belongs to class II, and PtmT1 from *Streptomyces platensis* in the platensimycin gene cluster [20], which is neither characteristic for class I nor class II. In fungi and plants, they can be both class I or class II TPSs and even mixed class I/II [21]. Class I TPSs harbor two distinct catalytic motifs, which are crucial for binding and correctly positioning the substrate for catalysis, the aspartate-rich motif (DDxxD) and the NSE/DTE motif, that bind to three Mg^2+^ ions [1,22]. Notably, the PtmT1 diterpene synthase from *S. platensis* lacks both catalytic motifs [20,23]. The NSE/DTE motif illustrates the distribution of DTE motifs, which are predominantly found in plant TPSs, and NSE motifs, which are more common in bacterial and fungal TPSs. For the description of the bacterial TPS CotB2, we will therefore refer to the NSE motif only.

Using host microorganisms, such as bacteria or baker’s yeast for the heterologous synthesis of terpenes increases the sustainability of bioactive terpene production by saving resources, as the production host can be fed with residual organic waste streams, such as milling or forestry waste. Additionally, the heterologous terpene production minimizes waste generation as the targeted production of a single compound reduces extraction and purification steps [24–25]. Additionally, heterologous production enables protein engineering to optimize product ratios or to alter the native product portfolio of the enzyme [9,26–27]. Furthermore, production by engineered microorganisms considerably reduces the cost compared to total chemical synthesis or extraction from natural sources, since the target compounds are produced from inexpensive carbon sources. Prominent examples of optimized terpene production pathways in *E. coli* are taxadiene, a precursor of the anticancer drug taxol [28], amorpha‐4,11‐diene, an antimalarial drug precursor [29], and cyclooctatin [30].

The scope of this review encompasses a detailed consideration of the cyclooctatin biosynthetic gene cluster in particular the TPS CotB2 from the soil bacterium *Streptomyces melanosporofaciens* MI614-43F2 (Figure 1) [31]. Cyclooctatin **5**, with its distinct 5–8–5 ring motif, belongs to the fusicoccane diterpenoids that encompass a wide range of bioactivities, such as bacteriostatic, fungicidal and tumorstatic effects [32]. A key player in the biosynthesis of cyclooctatin **5** is the bacterial diterpene synthase CotB2.

Different research teams have investigated CotB2 by means of biochemical [30–31,38], biophysical [33–35], structural biology [36–39] and computational modeling approaches [35,37,39–40], revealing a rather unusual reaction mechanism. Given the diverse bioactivities of cyclooctatin **5**, CotB2 is of biotechnological relevance as well. Cyclooctatin **5** can be manufactured biotechnologically in *E. coli* in milligram scales [26,30], which is important for an industrial application. Within the subsequent paragraphs, we would like to summarize the current understanding of the unusual reaction mechanism and the biotechnological applications.

## Review

### The cyclooctatin biosynthetic gene cluster

The cyclooctatin gene cluster from the soil bacterium *S. melanosporofaciens* MI614-43F2 comprises four enzymes: GGDP synthase (CotB1), a diterpene cyclase (CotB2) and two P450 cytochromes (Figure 1) [31]. The cyclization of GGDP **3** to cyclooctat-9-en-7-ol (**4**) is performed by the TPS CotB2. Compound **4** is further decorated with two hydroxy functions introduced by two P450 cytochromes, CotB3 and CotB4, to cyclooctatin (**5**). Depending on the functionalization of the ring system, the compounds demonstrate a broad diversity of biological activities, among others fungicidal or tumor static [32,43–45]. Cyclooctatin (**5**) inhibits a lysophospholipase, which catalyzes the hydrolysis of the fatty acid ester bonds of lysophospholipids [31,41]. Moreover, cyclooctatin (**5**) was shown to be effective against *Plasmodium falciparum* with an IC_50_ of 7.14 µg/mL along with very low cytotoxicity [42].

CotB2 belongs to the class of cyclopentane-forming diterpene synthases [46], a class of enzymes that is widely distributed among plants, fungi and bacteria. CotB2 has evolved to convert the acyclic, achiral substrate GGDP to the 5–8–5 ring motif of cyclooctat-9-en-7-ol that contains six chiral stereocenters. Hence, CotB2 has been fine tuned to perform a highly specific regio- and stereochemical reaction. The cyclization mechanism of CotB2 has been investigated extensively in recent years. By isotope labeling and NMR spectroscopic investigations [35], it has been shown that CotB2 catalyzes the complex regio- and stereospecific cyclization reaction with an unusual carbon–carbon bond rearrangement. Hong and Tantillo performed the first theoretical study of the reaction mechanism in CotB2 via gas phase density functional theory (DFT) calculations [33]. Simultaneously, Sato et al. also studied the CotB2 reaction mechanism in the gas phase using DFT, combined with experimental deuterium labeling [34]. Another important step towards a deeper understanding of the cyclization mechanism was the availability of crystal structures (Table 1) revealing structural snapshots along the reaction trajectory commencing with the open, inactive conformation completing with the closed, active conformation of CotB2. [36–38].

**Table 1:** Overview of available crystal structures of CotB2 and its variants.

protein/variant	PDB-ID	conformation	ligands	reference

CotB2^wt^	4OMG	open	–	[38]
CotB2^wt^	5GUC	open	–	[36]
CotB2^F149L^	4OMH	open	–	[38]
CotB2^ΔC^	6GGK	open	–	[37,39]
CotB2^F107A^·Mg^2+^_B_	6GGL	intermediate	1 Mg^2+^	[37,39]
CotB2^wt^·Mg^2+^_B_·GGSDP	5GUE	intermediate	1 Mg^2+^, geranyl geranyl thiodiphosphate (GGSDP)	[36]
CotB2^wt^·Mg^2+^_3_·F-Dola	6GGI	closed	3 Mg^2+^, diphosphate, 2-fluoro-3,7,18-dolabellatriene	[37,39]
CotB2^wt^·Mg^2+^_3_·AHD	6GGJ	closed	3 Mg^2+^, 4-amino-1-hydroxy-1-phosphonobutyl phosphonic acid	[37,39]

### Overall structure of CotB2^wt^ in the open, inactive conformation

**Figure 2:**
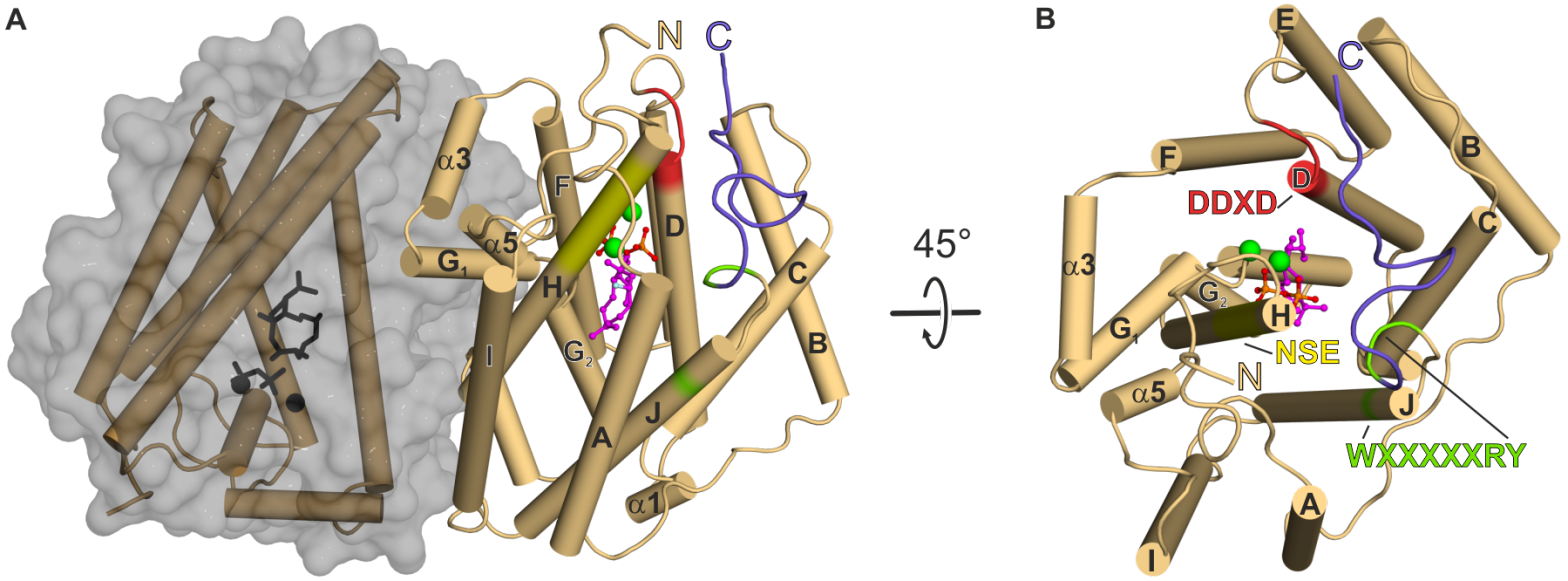
The bacterial diterpene synthase CotB2^wt^·Mg^2+^_3_·F-Dola in the closed, active conformation (PDB-ID 6GGI; [37]). (A) The two monomers of CotB2 are shown in cartoon representation with the α-helices drawn as cylinders and colored in light brown. One monomer of CotB2 is shown in gray surface representation. The location of the aspartate-rich motif is indicated in red and the NSE-motif is marked in yellow. The WXXXXXRY motif is indicated in light-green. The last 12 C-terminal residues of the lid are drawn in violet. The three Mg^2+^ ions are represented as green spheres and the bound intermediate F-Dola is shown in magenta. The cleaved diphosphate is shown in orange. (B) View of panel A rotated by 45°. For clarity, one monomer has been removed. The view is into the active site of CotB2.

The structure of CotB2^wt^ (PDB-ID 4OMG [38] and PDB-ID 5GUC [36]) is complete, except for the 15 N-terminal and 12 C-terminal residues. CotB2 consists of ten core α-helices (A to J) that are connected by short loop segments and additional five short α-helices (α1 to α5; (Figure 2A and B)). CotB2 resembles the classical α-helical fold of TPSs [18] with significant differences in its primary sequence compared to other TPSs. The core α-helices surround a large, deep cleft, which forms the active site (Figure 2A and B). CotB2 is arranged as a homo-dimer in crystallo [36,38] and it was demonstrated that CotB2 exists as a homo-dimer in solution as well [38]. The two active sites of CotB2 are arranged in an antiparallel fashion, resembling the arrangement initially observed for the monoterpene (+)-bornyl diphosphate synthase [47] and the sesquiterpene trichodiene synthase [48], but is in contrast to the parallel dimer as described for farnesyl diphosphate synthase [49]. Structurally, CotB2 is most closely related to the fungal monoterpene synthase aristolochene (PDB-ID 2OA6; [50]), and epi-isozizaene synthase (PDB-ID 3LGK; [51]), but not to the known plant structures of diterpene TPSs, such as the ent-copalyl diphosphate synthase (PDB-ID 3PYA; [52]) or taxadiene synthase (PDB-ID 3P5R; [53]).

### Two crystal structures of CotB2 resembling two distinct precatalytic states

The structure of CotB2^F107A^ was determined with one single Mg^2+^ (Mg^2+^_B_) bound to the NSE motif (CotB2^F107A^·Mg^2+^_B_; PDB-ID 6GGL [37]). The overall structure of CotB2 remains unchanged compared to the open state. This suggests that binding of Mg^2+^_B_ is the initial step to prearrange CotB2 for substrate binding.

**Figure 3:**
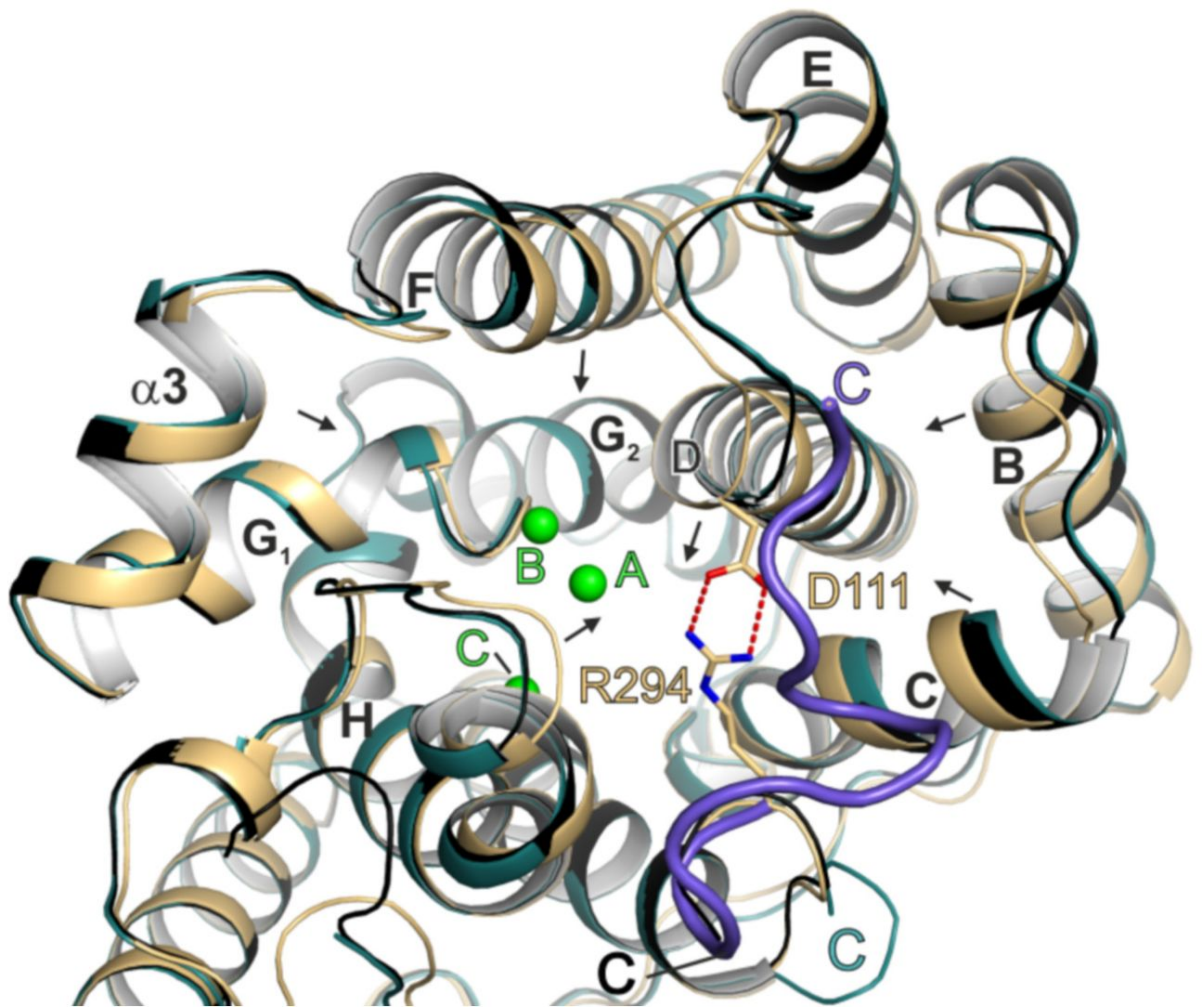
Conformational changes of CotB2 upon ligand binding. Superposition of CotB2’s open (teal), pre-catalytic (black, CotB2^wt^·Mg^2+^_B_·GGSDP), and fully closed (light-brown, CotB2^wt^·Mg^2+^_3_·F-Dola) conformation. The overall fold of CotB2^wt^·Mg^2+^_B_·GGSDP (PDB-ID 5GUE; [36]) is more similar to CotB2^wt^ (PDB-ID 4OMG; [38]) than to CotB2^wt^·Mg^2+^_3_·F-Dola (PDB-ID 6GGI; [37]). The salt bridge between D111 and R294, in stick representation, is shown by red, dashed lines. Mg^2+^ ions are colored in green. Black arrows indicate movement of secondary structure elements from the open to the closed conformation of CotB2. The different C-termini are labeled in all three structures. The C-terminus in the structure of CotB2^wt^·Mg^2+^_3_·F-Dola is colored in purple.

Tomita et al. obtained a crystal structure of CotB2^wt^ with bound inert substrate analogue geranylgeranyl thiodiphosphate (GGSDP; CotB2^wt^·Mg^2+^_B_·GGSDP; PDB-ID 5GUE; [36]), resembling the next precatalytic state of CotB2. Surprisingly, there is only Mg^2+^_B_ bound to the enzyme and not the full set of three Mg^2+^-ions needed for catalysis. The position of Mg^2+^_B_ is identical to the structure of CotB2^F107A^·Mg^2+^_B_, but two coordinating water molecules have now been exchanged with two oxygen atoms of the diphosphate function of GGSDP. Upon binding of GGSDP, a bidentate salt bridge between D111 of the DDXD motif and R294 of the WXXXXXRY motif is formed, designating the transition from the open to the closed conformation. However as for the open, inactive conformation, the remaining C-terminal residues have not been traceable in the electron density (Figure 3), and hence CotB2 is not yet ready for catalysis. The position and tilting of the core α-helices in the structure of CotB2^wt^·Mg^2+^_B_·GGSDP is practically indistinguishable from the open conformation of CotB2 (Figure 3). Since in CotB2^wt^·Mg^2+^_B_·GGSDP two Mg^2+^ ions are absent the diphosphate moiety is not properly coordinated, resulting in significant substrate flexibility within the catalytically active site. Moreover, the two missing Mg^2+^ ions prevent helix D, accommodating the Asp-rich motif, from shifting towards the active site (Figure 3). Consequently, the active site remains partially open and the C-terminus cannot fold over the active site, making a proper substrate positioning and subsequent cyclization unlikely.

### Crystal structure of CotB2^wt^·Mg^2+^_3_·F-Dola resembles the closed, active conformation

The structure of CotB2^wt^·Mg^2+^_3_·F-Dola has been obtained by co-crystallization of CotB2^wt^ and FGGDP (PDB-ID 6GGI; [37]), representing the closed, active conformation. Fluorinated substrates, such as FGGDP, have been used for the crystallization of other TPSs and have been shown to stick to the active site without undergoing cyclization, trapping the enzyme in a closed state [52–53]. Comparing the overall structure of CotB2^wt^·Mg^2+^_3_·F-Dola to the open conformation of CotB2 reveals major differences. The binding of FGGDP and three Mg^2+^ ions induces a translation and rotation of α-helices B, C, D, F and H towards the active site to accurately position the Asp-rich motif and to subsequently transfer the active site into a product-shaped conformation (Figure 3). Now, a significant change in the C-terminal region of the enzyme is observed as the former unstructured C-terminus becomes folded, thereby acting as a lid to shield the active site from bulk solvent (Figure 2). To exclude that the folded C-terminus is due to crystal contacts and hence a crystallographic artifact, CotB2 was co-crystallized with AHD, a compound that mimics the diphosphate group of GGDP and acts as a suicide inhibitor. The crystal structure of CotB2^wt^·Mg^2+^_3_·AHD (PDB-ID 6GGJ; [37]) confirmed the active site architecture as observed in the structure of CotB2^wt^·Mg^2+^_3_·F-Dola and more importantly, that the folding of the C-terminus is not a crystallographic artifact. To demonstrate the significance of the C-terminus, a CotB2 truncation (CotB2^ΔC^) was designed terminating at R294, thereby missing the 12 C-terminal residues, that correspond to the lid [37]. In activity experiments, no substrate conversion by CotB2^ΔC^ was observed, stressing the fact, that the salt bridge D111-R294 is insufficient for catalysis. Instead, the full C-terminus is required for catalysis.

**Figure 4:**
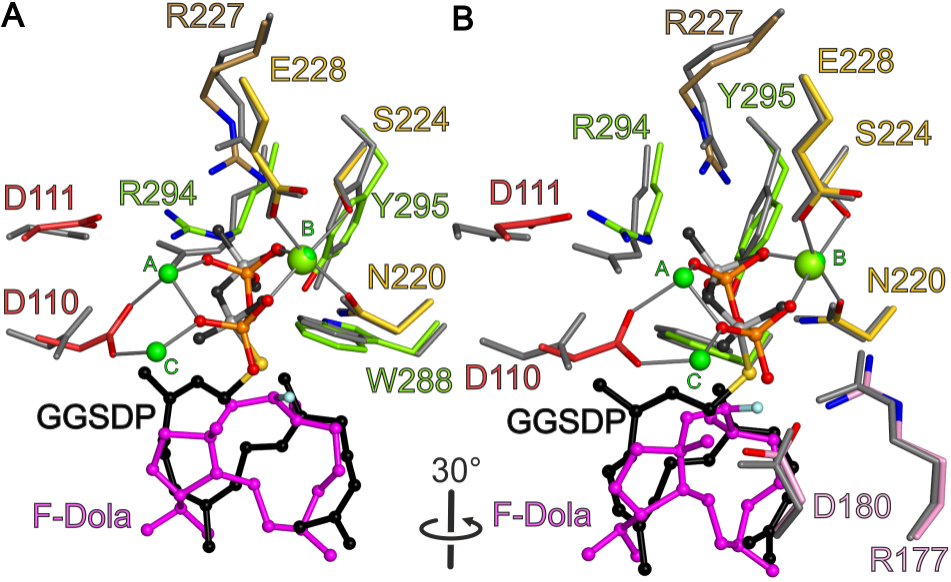
View into the active site of CotB2^wt^·Mg^2+^_3_·F-Dola [37] superimposed with CotB2^wt^·Mg^2+^_B_·GGSDP [36]. (A) The bound F-Dola reaction intermediate is shown in magenta with the fluorine atom colored in light blue, and Mg^2+^ions are shown in green. Residues of the DDXD motif are shown in red and residues of the NSE motif in yellow. The conserved residues of the WXXXXXRY motif are drawn in light green and the R227 that interacts with diphosphate moiety in light brown. The cleaved diphosphate is shown in orange. Gray lines represent the coordination sphere of the Mg^2+^ ions. Identical amino acid residues located in the structure of CotB2^wt^·Mg^2+^_B_·GGSDP are shown in gray. The GGSDP molecule is shown in black with the sulfur atom colored in yellow. The position of Mg^2+^_B_ is identical in both structures. For clarity water molecules have been omitted. (B) View in panel A rotated by 30°. In addition to the motifs shown in panel A, the pyrophosphate sensor motif is depicted in pink.

In CotB2^wt^·Mg^2+^_3_·F-Dola, there is no electron density observed for the intact FGGDP molecule. Instead two distinct and clearly separated electron densities are visible that could be readily interpreted as the reaction intermediate F-Dola, as well as a single diphosphate moiety. The FGGDP molecule – functionalized with a fluorine atom at the C2 position of its isoprenoid unit – can interfere with the propagation of the generated carbocation(s). Such cyclization reactions of fluorinated substrates have been reported previously [54–55]. However, the structure of CotB2^wt^·Mg^2+^_3_·F-Dola represents the first reported structure of a TPS with an in crystallo formed and bound reaction product, together with the cleaved-off diphosphate. This is in contrast to the structure of taxadiene synthase (PDB-ID 3P5R; [53]), co-crystallized with FGGDP, where the substrate is bound but the cyclization reaction has not been initialized and consequently the substrate is not yet cleaved. Identical observations have been made for the crystal structure of aristolochene synthase co-crystallized with 2-fluoro diphosphate (PDB-ID 3BNY; [56]). A structural comparison of CotB2^wt^·Mg^2+^_3_·F-Dola and CotB2^wt^·Mg^2+^_B_·GGSDP reveals that the hydrophobic tail of GGSDP adopts a position similar to the cyclized intermediate in our CotB2^wt^·Mg^2+^_3_·F-Dola structure (Figure 4), likely reflecting the already product-shaped architecture of the active site.

### Catalytic motifs of CotB2

#### Mg^2+^ coordination by the aspartate-rich and NSE motif

**Figure 5:**
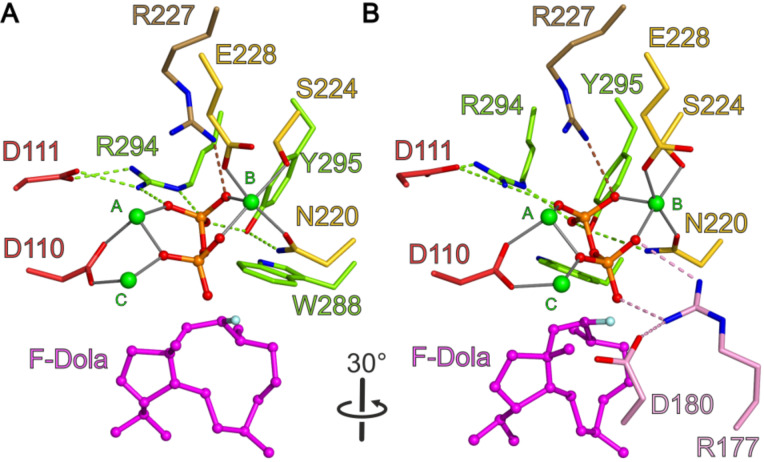
View into the active site of CotB2^wt^·Mg^2+^_3_·F-Dola [37]. Identical view as in Figure 4. (A) The bound F-Dola reaction intermediate is shown in magenta with the fluorine atom colored in light blue, and Mg^2+^-ions are shown in green. Residues of the DDXD motif are shown in red and residues of the NSE motif in yellow. The conserved residues of the WXXXXXRY motif are drawn in light green and the R227 that interacts with diphosphate moiety in light brown. The cleaved diphosphate is shown in orange. Hydrogen bonds and salt bridges are indicated by dashed lines in the same color-coding as the involved motifs. Gray lines represent the coordination sphere of the Mg^2+^-ions. For clarity water molecules have been omitted. (B) View in panel A rotated by 30°. In addition to the motifs shown in panel A, the pyrophosphate sensor motif is depicted in pink.

Instead of the classical DDxxD motif for class I TPS, CotB2 contains a modified ^110^DDxD^113^ motif, which is neither characteristic for class I nor class II terpene synthases. In the literature, other TPSs with modified aspartate‐rich motifs have been reported, encompassing selina‐3,7(11)‐diene synthase: ^82^DDGYCE^87^ [57] and (+)‐T-muurolol synthase: ^83^DDEYCD^88^ [58]. It is commonly observed that the aspartate-rich motif resides at the lower part of α-helix D in the structure of class I TPSs. Interestingly, in CotB2, α-helix D is rather short as a proline residue, adjacent to the third aspartate of the aspartate-rich motif, introduces a kink. In the closed conformation D110 is directly involved in the coordination of Mg^2+^_A_ and Mg^2+^_C_, whereas D111 forms a salt bridge with R294 (Figure 5). A third Mg^2+^ (Mg^2+^_B_) is bound by the residues N220, S224 and E228 of the NSE motif [59], that is located on α-helix E of CotB2 (Figure 5). D113 is surface exposed and points away from the active site and hence is likely not be involved in catalysis. Latter structural findings are supported by site-directed mutagenesis studies [36,38], which showed that, whereas D110 and D111 are crucial for catalysis, the solvent exposed D113 is not. The here described structural situation is different compared to TPSs with a canonical DDXXD motif. For instance in the structure of epi-isozizaene synthase in complex with diphosphate, 3 Mg^2+^ ions and *N*-benzyl-*N*,*N-*diethylethanaminium [51], the α-helix is longer. Compared to CotB2, the first aspartate is as well involved in the coordination of Mg^2+^_A_ and Mg^2+^_C_ and the second aspartate forms a salt bridge with the RY-pair, but the third aspartate is involved in coordination of water molecules in the water network around the catalytic Mg^2+^ ions.

#### The pyrophosphate sensor motif

In a recent review, the presence of a “pyrophosphate sensor”, an arginine 46 amino acid residues upstream of the NSE motif, was discussed as a universal feature of bacterial TPSs [11]. Structural information on selina-4(15),7(11)-diene synthase (SdS), revealed that upon substrate binding by an induced fit mechanism, R178 changes its side chain conformation thereby approaching and interacting with the diphosphate function of the substrate analogue and forming a salt bridge to the neighboring D181 [60]. Moreover, a conformational change of the kink of the α-helix G1 moves the catalytically important G182 (effector) towards the active site. Initiated by this induced fit mechanism, the active site is being closed and the Michaelis complex is formed. In CotB2, R177 represents the pyrophosphate sensor (Figure 5B) establishing identical interactions to the diphosphate moiety of the substrate and to D180. In contrast to SdS, in CotB2 an additional residue is inserted between D180 and the effector G182. A comparison of the open and closed conformation of SdS revealed a large conformation change of the G1/G2 helix kink, which is not observed in the structures of CotB2 [36–38]. This might be explained by the observation that the pyrophosphate sensor of CotB2 R177 and D180 is already involved in a salt bridge in the open conformation.

#### The WXXXXXRY motif

**Figure 6:**
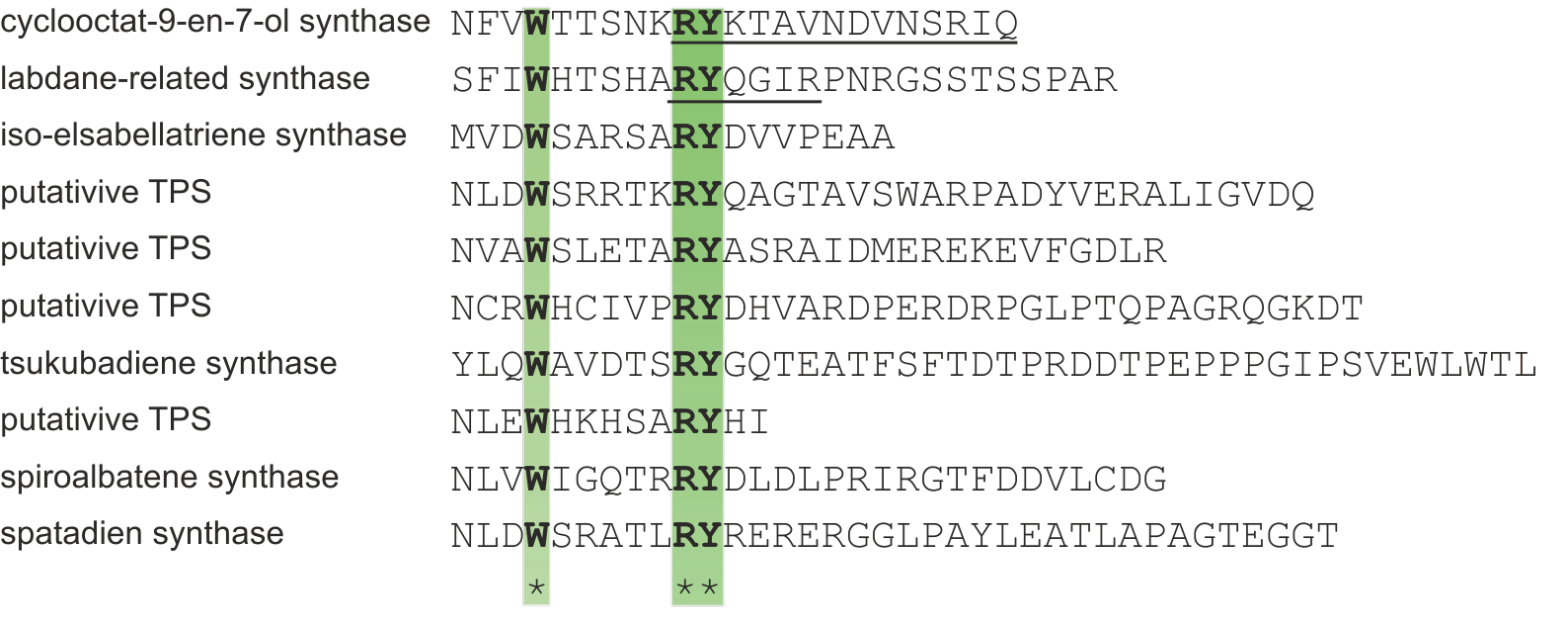
The WXXXXXRY motif in protein sequences of diterpene TPS from different bacteria. Highlighted is the WXXXXXRY motif in green. Underlined sequences refer to crystal structures of the respective diterpene TPS in the closed conformation, with a structured C-terminus. Cyclooctat-9-en-7-ol synthase (WP_093468823), *S. melanosporofaciens;* labdane-related diterpene synthase (WP_019525557), *Streptomyces* K155; iso-elsabellatriene synthase (WP_003963279), *Streptomyces clavuligerus*; terpene synthase (WP_046708564.1), *Streptomyces europaeiscabiei*; terpene synthase (WP_012394883), *Myobacterium marinum*; diterpene synthase (BAP82229), *Streptomyces* sp. ND90; tsukubadiene synthase (EIF90392), *Streptomyces tsukubaensis* NRRL 18488; terpene synthase (ZP_00085244), *Pseudomonas fluorescens* PfO-1; spiroalbatene synthase (WP_030426588.1), *Allokutzneria albata*; spatadien synthase (WP_095757924.1) *Streptomyces xinghaiensis*.

Analyzing protein sequences of other bacterial TPSs for the presence of the RY pair, also called “basic pair” [61–62] and their flanking regions, led to the identification of a conserved tryptophan six amino acids upstream of the RY pair (Figure 6) [37]. In CotB2, residues of the motif are located at the end of the C-terminal core α-helix J. The RY pair is a conserved feature among most bacterial and several types of fungal TPSs [62]. It is not only important for active site closure by establishing a salt bridge, but moreover for interaction with the substrate. In the closed conformation of CotB2, R294 is involved in a bidentate salt bridge with D111 of the aspartate-rich motif, and two salt bridges to the diphosphate moiety derived from FGGDP (Figure 3 and Figure 5). Y295 establishes hydrogen bonds to the diphosphate moiety as well as to N220 of the NSE motif (Figure 5). In the closed conformation of the labdane-related diterpene synthase [63] the WXXXXXRY motif is present as well and adopts a nearly identical conformation as observed in the closed conformation of CotB2^wt^·Mg^2+^_3_·F-Dola (PDB-ID 6GGI; [37]). Hence, the WXXXXXRY motif seems to be conserved not only sequence-based (Figure 6) but also structurally in other diterpene synthases. Hence, the discovery of this novel motif might help in the identification and functional assignment of novel TPSs.

The importance of the tryptophan residue in the WXXXXXRY motif of CotB2 was proven by mutation to glycine (W288G), where the product was changed to 3,7,18-dolabellatriene (**12**) [38]. If a less drastic mutation was introduced (W288F), preserving the aromatic character of the side chain, the product was not changed but the activity of CotB2 was reduced [37]. In other TPSs, a similar observation was made. Mutation of the corresponding residue W308F in the bacterial sesquiterpene synthase pentalenene synthase leads to a product mixture [64], whereas mutation of W273 in the plant-derived epi-aristolochene synthase resulted in total loss of enzymatic function [65]. Consequently, the aromatic character of the side chain in the WXXXXXRY motif is important for the propagation of the carbocation.

#### Mechanistic aspects of the cyclization reaction and point mutations

**Scheme 1:**
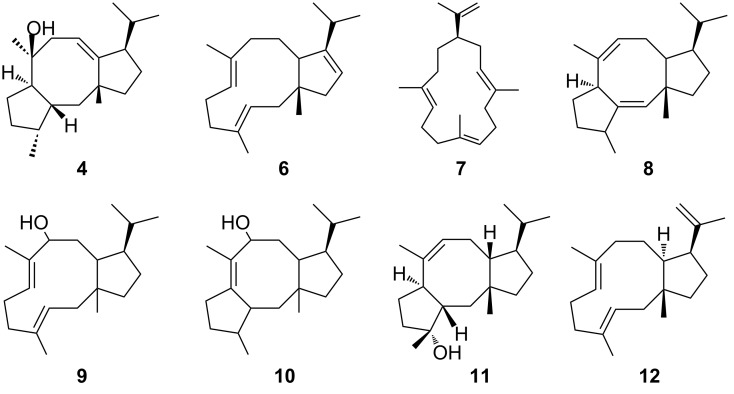
Overview of the altered product portfolio as a result of introduced point mutations in the active site of CotB2. For an overview of the point mutations see Table 2.

**Scheme 2:**
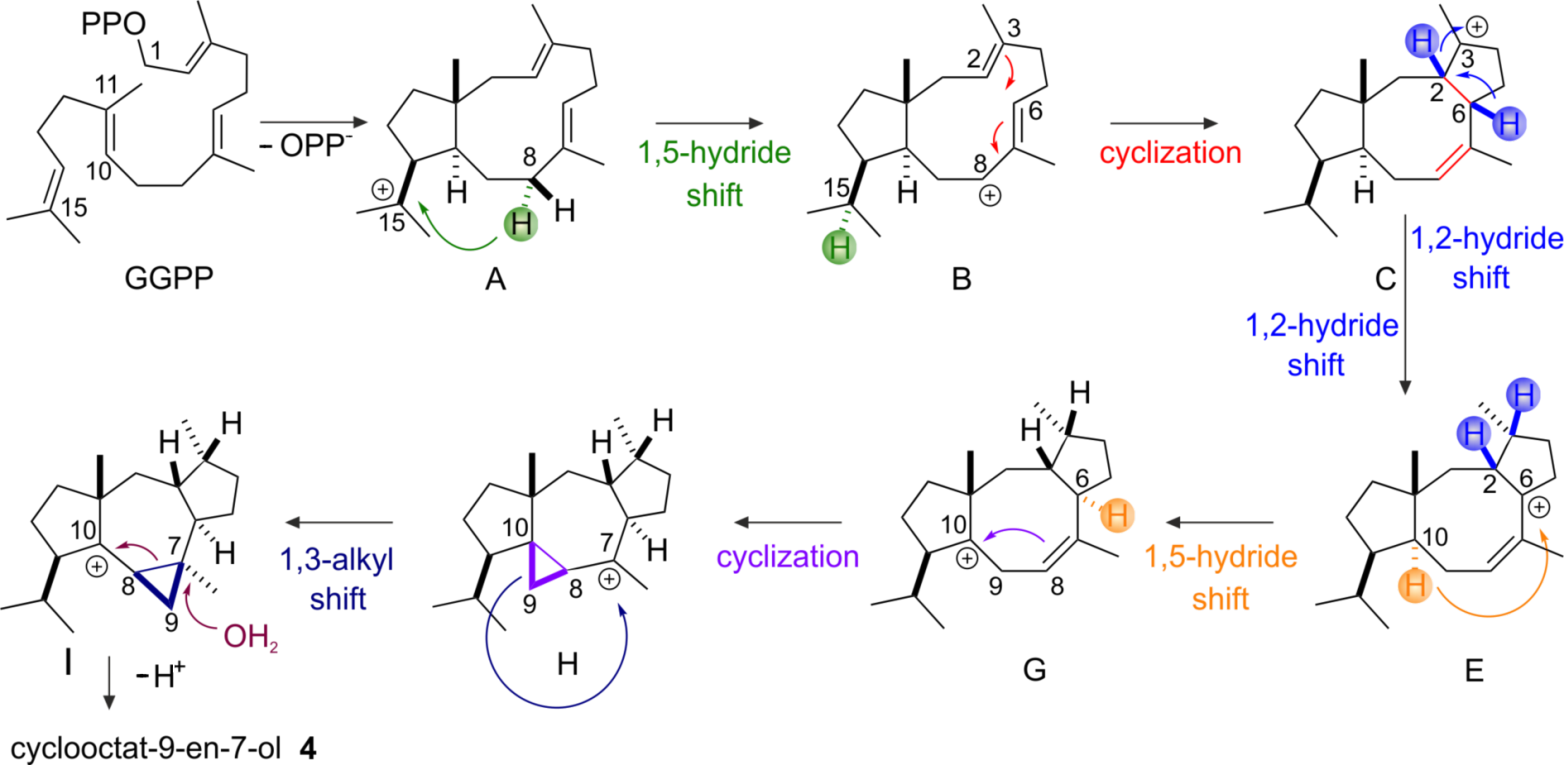
Catalytic mechanism of CotB2, derived from isotope labeling experiments [34–35], density functional theory calculations [33] as well as QM/MM simulations [37].

The floor of the active site is decorated by hydrophobic residues V80, F107, W109, F149, V150, I181, F185, M189, W186, L281, and W288 conferring the overall shape to the cavity that serves as a template for the binding of GGDP and the cyclization reaction. Based on ^2^H- as well as ^13^C-isotope labeling experiments a surprising reaction mechanism has been derived (Scheme 2) [35]. Strong support for the proposed reaction mechanism has been predominantly provided by site-directed mutagenesis of amino acids with an aromatic function [30,36–38] (Table 2 and Scheme 1), which significantly affect the product profile.

**Table 2:** CotB2 and its variants as well as the altered products.

variant	product	reference

wild-type	cyclooctat-9-en-7-ol (**4**)	[31]
N103A	3,7,12-dolabellatriene (**6**)	[36]
F107A	*R*-cembrene A (**7**)	[30]
F107Y	cyclooctat-1,7-diene (**8**)	[30]
F107L	cyclooctat-9-en-7-ol (**4**) 3,7-dolabelladiene-9-ol (**9**) cyclooctat-6-en-8-ol (**10**)	[30,36]
F149L	cyclooctat-7-en-3-ol (**11**)	[30]
F185A	cyclooctat-9-en-7-ol (**4**) cyclooctat-6-en-8-ol (**10**)	[36]
W186L	cyclooctat-9-en-7-ol (**4**) cembrane A (**7**) 3,7,18-dolabellatriene (**12**)	[36]
W186F	cyclooctat-9-en-7-ol (**4**) cyclooctat-7-en-3-ol (**11**) 3,7-dolabelladiene-9-ol (**9**) cyclooctat-6-en-8-ol (**10**)	[36]
W186H	cyclooctat-7-en-3-ol (**11**) 3,7,18-dolabellatriene (**12**)	[36,38]
W288G	3,7,18-dolabellatriene (**12**)	[38]

Further support for the reaction mechanism was generated by gas-phase calculations [33–34] as well as in silico multiscale modeling [37], which suggest an active role of the enzyme during catalysis.

**Figure 7:**
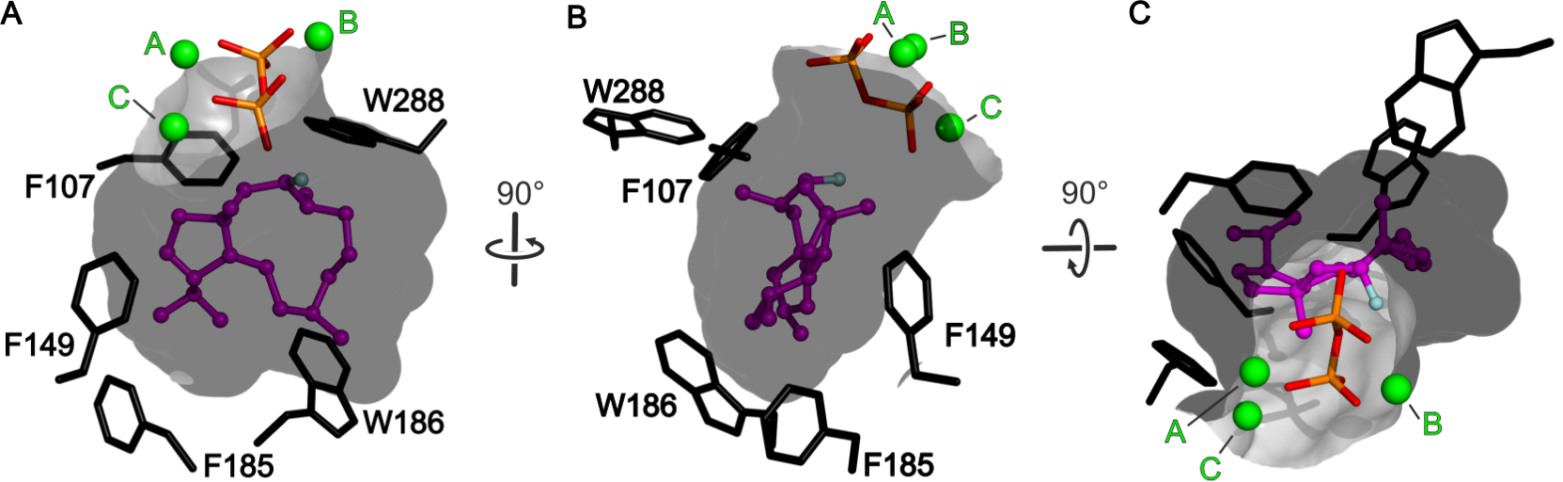
(A) The inner surface of the active site is shown in gray. The bound F-Dola reaction intermediate is shown in magenta with the fluorine atom colored in light blue. Aromatic residues are shown in black stick representation (B) View of panel A rotated by 90°. (C) View from the top into the active site of CotB2. View of panel A rotated by 90°.

Cyclization is initiated by cleavage of the GGDP diphosphate moiety. After two consecutive cyclization reactions, a dolabellatrienyl cation (**A**) is generated, stabilized by π-cation interactions with W186 (Scheme 2 and Figure 7). Whereas mutation of this residue to amino acids with aromatic character mainly lead to different migration of the double bound and different hydroxylation pattern (Table 2 and Scheme 1). An exchange to leucine drastically changes the product to cembrane A (**7**) and 3,7,18-dolabellatriene **12** (Table 2 and Scheme 1) [36]. The cation migrates via a 1,5 hydride shift, as shown by deuterium labeling [33–35], to form the carbocation located at position C8 of dolabellatrienyl (**B**). By ring closure and formation of a novel C–C bond the tricyclic 5–8–5 ring system (**C**) is established. F107, F149, and the pyrophosphate moiety stabilize the carbocation at position C3 (Scheme 2 and Figure 7). F107 has been targeted by site-directed mutagenesis as well (Table 2 and Scheme 1) [30], leading to compounds *R*-cembrene A (**7**) and cyclooctat-1,7-diene (**8**). Now the cationic intermediate has two possibilities to react to **G**, either by a 1,3- and 1,5-hydride shift or by two 1,2-hydride shifts followed by a 1,5-hydride shift. The sequential 1,2-hydride shift (**C** to **E**) route was initially suggested by theoretical calculations, which supported the overall mechanism [33–34], and this was verified via isotope labeling [34]. Such series of two 1,2-hydride shifts have previously been demonstrated experimentally for tsukubadiene synthase [66]. Compound **E** is stabilized by F107 (Scheme 2 and Figure 7) and the backbone carbonyl of I181. Compound **G** is obtained by another 1,5-hydride shift as proven by isotope labeling [35]. The carbocation located at C10 is stabilized by N103 and T106. A mutation of N103 to alanine, bearing the possibility to stabilize the carbocation, results in 3,7,12-dolabellatriene **6** (Table 2 and Scheme 1). Finally, the cyclopropyl ring is opened by a 1,3-alkyl shift and a subsequent nucleophilic attack by a water molecule at C7 yields the product cycloocat-9-en-7-ol (**4**). Notably, no water has been observed in the structural investigations that could resume the nucleophilic attack.

#### Biotechnological applications to exploit the chemistry of CotB2

The mutants described in Table 2 are not only important for resolving structure–function relationships, they also convey the conversion to carbon skeletons of other bioactive natural product classes. In light of this fact, the exchange of only one amino acid changes the product portfolio from cyclooctatin (**5**) belonging to the fusicoccane diterpene family to compounds of the dolabellane [67] or the cembranoid class [68].

**Scheme 3:**
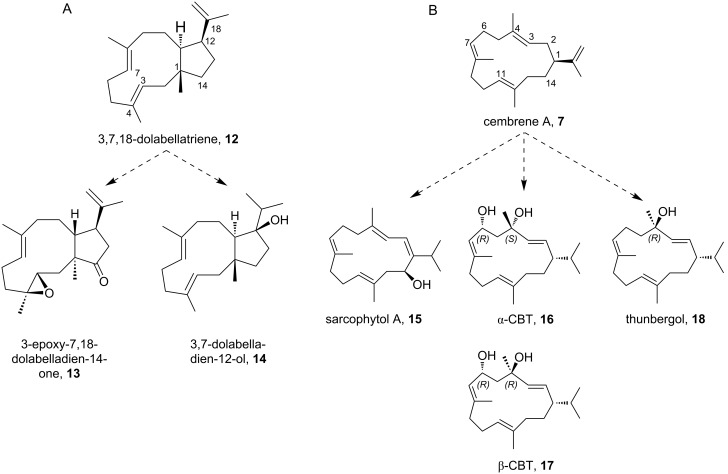
Variants of CotB2 open the route to a novel product portfolio with altered cyclic carbon skeletons, which can be converted into bioactive compounds by chemo-enzymatic methodologies. Modification descriptions are composed according to the atom numbering. (A) Lead structure for the dolabellane derivatives is 3,7,18-dolabellatriene (**12**) and (B) cembrene A (**7**) for the cembranoid family.

The dolabellane diterpenes synthesized by the respective synthase mutants are missing the bond between C2 and C6 compared to cyclooctatin (**5**). Hence, they are comprising a 5,11-fused bicyclic skeleton. In contrast, cembranoids are monocyclic compounds consisting of a 14 membered ring structure. Common to all compounds available via the CotB2 mutants is the necessity of further decoration with functional groups, like hydroxylations or epoxidations, to induce the desired bioactivity (Scheme 3). Modification descriptions in the next chapters are composed according to the atom numbering in (Scheme 3). Lead structure for the dolabellane derivatives is 3,7,18-dolabellatriene (**12**) and cembrene A (**7**) for the cembranoid family, respectively (Scheme 3).

Dolabellanes comprise a diverse group of bioactive diterpenes, with the first compounds initially isolated from the mollusc *Dolabella californica* in 1977 [69]. Later, the product family was widened by molecules isolated from other marine organisms, like sponges, sea whips and the brown algae of this genus Dictyota [70]. Two modifications would transform 3,7,18-dolabellatriene (**12**, Scheme 3) into a potent cytotoxic molecule with activity towards murine leukemia cells or human non-small cell lung cancer with ED_50_ values of 6.5 and 16.7 µg mL^−1^, respectively [71]. To obtain this interesting compound isolated from *Dictyota dichotoma*, C14 has to be modified with a keto function and the double bond between C3 and C4 has to be converted into an epoxide. Additionally, 3,7,18-dolabellatriene (**12**) possesses a mainly weak antibiotic activity against different types of *Staphylococcus aureus* strains (two epidemic MRSA strains, a macrolide-resistant variant and two multidrug-resistant efluxing strains) [72]. By contrast, 3,7,18-dolabellatriene (**12**, Scheme 3), only altered at C14 with a hydroxy group, showed a significant increase in potency (compound **14**). Here the MIC values were as low as 4 µg mL^−1^ for a macrolide-resistant and one multidrug-resistant effluxing variant, as well as 2 µg mL^−1^ for two epidemic MRSA and another multidrug-resistant effluxing strain.

The first bioactive molecule of the cembranoid family sarcophytol A (**15**), isolated in 1979 from the soft coral *Sarcophyton glaucum* [73], is structurally closely related to *R*-cembrene A (**7**, Scheme 3). In order to obtain sarcophytol A (**15**), two reactions have to be applied to compound **7**: First a base induced double bond shift to gain cembrene C and second a hydroxylation at C14. Sarcophytol A (**15**), is a promising target as it possesses inhibitory activity against potent tumor promotors, like teleocidin [74]. Interestingly, sarcophytol A (**15**) was already inhibitory at equimolar amounts in contrast to other natural antitumor promotors, like flavonoids or dihydroxycembranoids from tobacco leaves, which has to be applied at a factor of thousand or more compared to the promotor quantity to be reasonably active. Moreover, sarcophytol A (**15**) inhibits the expression of the tumor necrosis factor-α (TNF-α) mRNA and the release of TNF-α by BALB/3T3 cells [75]. This cancer prevention activity can be initiated at an IC_50_ of only 2.5 µM.

Other bioactive target molecules structurally accessible from compound **7** could be both epimers of (6*R*)-2,7,11-cembratriene-4,6-diol either 4S: α-CBT **16** or 4R: β-CBT **17** (Scheme 3). Both were isolated in 1985 from cigarette smoke condensate and identified as anticancer agents [76]. With quite similar potency (α-CBT: 25.2 µM and β-CBT: 21.9 µM [77]), they showed inhibition of the induction of Epstein–Barr virus early antigen by lymphoblastoid cancer cells. In the tobacco plant itself, both substances, in addition to being key flavor ingredients [78], play a major role in the defence against insects, pathogenic microbes and herbivores [79]. Furthermore, β-CBT **17** showed additional antibiotic activity against multidrug resistant *S. aureus* strains, [80], as well as neuroprotective activity [81].

Interestingly, the β-CBT derivative missing the hydroxy group at C6 called thunbergol (**18**, Scheme 3), already inhibited the growth of two parasites *Trypanosoma brucei rhodesiense*, causing African sleeping sickness, as well as *Plasmodium falciparum* causing Malaria tropica, while having only minor human cytotoxicity [82]. Further, thunbergol (**18**) is reported to repel aphids within 48 h by 70%, if wheat seedlings are topically treated with a 0.25% (w/w) solution in ethyl acetate, compared to untreated plants [25]. During application in agriculture a negative impact on useful insects, like bees, should be avoided. A hint in this direction could be that in an in vitro experiment with *Spodoptera frugiperda* insect cells, a high resilience could be detected with an IC_50_ of 68 µM. Furthermore, Gram-positive bacteria are significantly growth inhibited through thunbergol (**18**) exposure with an IC_50_ for *Bacillus subtilis* of 9 µM and 10 µM for *Micrococcus luteus,* respectively [25].

For the sustainable, high yield production of bioactive diterpenoids various aspects have to be considered. One key issue in yielding high recombinant terpene production titers, are metabolic bottlenecks in the precursor supply, which have to be circumvented [83]. In that regard, there are different ways of enhancing *E. coli* host productivity. First, the general production pathway is usually genetically established. Choosing a plasmid-based production pathway has the advantage, that single genes can be exchanged or added quite rapidly [84]. Furthermore, decreasing the metabolic burden of the plasmid construct on the native host metabolism [85] can be achieved by using polycistronic operons to reduce the amount of plasmid in a cell. Additionally, computer aided fine-tuning [86] of transcription rates by promotor [24] and RBS [87] variations will further enhance the production rate [25]. Alternatively, permanent integration of the heterologous genes into the host genome is an alternative strategy to circumvent metabolic stress by antibiotics, which are required to maintain a plasmid in the production host [28]. Nevertheless, genomic integration procedures into the *E. coli* genome are time-consuming and have the disadvantage of having only a single copy in the host genome. Thus, expression and subsequently production rates of a previously optimized plasmid based system are not transferable to a genomically integrated system. In that respect, each operon has to be optimized de novo, whereby also the loci of integration have a severe impact on the expression rates of genome integrated heterologous gene material [88–90].

### Perspectives

CotB2 is an exciting example of how nature evolved an enzyme to perform very sophisticated chemistry. Mechanistically, CotB2 is very well understood, based on data from various different disciplines [30–31,33,35–38]. TPSs play an active role in all steps from the initialization of the diphosphate cleavage, and end with the nucleophilic attack of a water molecule. Yet, there is a dogmatic dispute in the TPS community about the respective roles of the protein acting as a scaffold and the “inherent” carbocation reactivity [91] in orchestrating an enzyme specific reaction cascade. With regard to the latter, the “inherent reactivity” of carbocations is no doubt an important ingredient in terpene biosynthesis. The enzyme could be understood as passive catalyst, essentially chaperoning the intermediates during the reaction cascade. It is clear that much of terpene biosynthesis can be understood by this concept. “Inherent reactivity” largely relies on theoretical calculations performed in the gas phase [92–93]. The latter approach per se disregards the role of the protein scaffold during catalysis. However, there are many examples of mono-, sesqui-, and diterpene synthases, where the contribution of the diphosphate and selected amino acid residues, which decorate the active site, on carbocation stability has been demonstrated [94–98]. These studies reveal a significant effect of individual active site moieties on TPS reactions in respect of product formation or alteration of the product profile. Additionally, numerous theoretical studies have emphasized the role of the enzyme environment in guiding the reaction cascade [94,99–102]. In case of CotB2, mutagenesis studies of plasticity residues of CotB2 [30,36–38] have been demonstrated to drastically change the propagation of the carbocations and consequently alter the product portfolio (Table 2 and Scheme 1).

Notably, in the crystal structures of CotB2 (Table 1) [36–38], we perceive that the amino acid side chains with an aromatic character in the active site (Figure 7) frequently adopt energetically unfavorable side chain conformations. Moreover, the crystal structures of the CotB2^F107A^ [37] and CotB2^F149L^ [38] variants revealed that other aromatic amino acid side chains adopt different side chain conformations compared to the structure of CotB2^wt^. This hints to long-range effects of the introduced mutation on other amino acid side chains in the active cavity. We are convinced that this plasticity of side chains in the active site of CotB2 plays not only an important role in stabilization and hence propagation of carbocations.

In future experiments, it would be interesting to investigate the influence of double or triple mutations within the active site. Moreover, it would be interesting to prepare a CotB2 variant with a canonical aspartate-rich motif and to study the influence on catalysis, whether the product portfolio is affected or not. Since until now no water molecule could be located in the electron density maps, in terms of catalysis the question remains which entity performs the nucleophilic attack at position C7 to give the final product cyclocotat-9-en-7-ol (**12**).

Another direction, to expand the chemical space, is the exploitation of TPSs to generate novel compounds that could be further functionalized by classical organic chemistry. Examples of this include epoxidation resulting in 3,4-epoxy-7,18-dolabelladien-14-one (**13**) or hydroboration of 3,7,18-dolabellatriene (**12**, Scheme 1) that has been previously biotechnologically manufactured using CotB2^W288G^ [103]. Another successful example is the oxidative transformation of cattleyene and phomopsen [104]. Yet another approach is the use of heteroatom-modified farnesyl diphosphates that could be still cyclized by TPSs yielding unnatural terpenoids [105].
